# Personality Consistency in Dogs: A Meta-Analysis

**DOI:** 10.1371/journal.pone.0054907

**Published:** 2013-01-23

**Authors:** Jamie L. Fratkin, David L. Sinn, Erika A. Patall, Samuel D. Gosling

**Affiliations:** 1 Department of Psychology, The University of Texas at Austin, Austin, Texas, United States of America; 2 School of Zoology, University of Tasmania, Hobart, Tasmania, Australia; 3 Department of Educational Psychology, The University of Texas at Austin, Austin, Texas, United States of America; Institute of Biology, University Leipzig, Germany

## Abstract

Personality, or consistent individual differences in behavior, is well established in studies of dogs. Such consistency implies predictability of behavior, but some recent research suggests that predictability cannot be assumed. In addition, anecdotally, many dog experts believe that ‘puppy tests’ measuring behavior during the first year of a dog's life are not accurate indicators of subsequent adult behavior. Personality consistency in dogs is an important aspect of human-dog relationships (e.g., when selecting dogs suitable for substance-detection work or placement in a family). Here we perform the first comprehensive meta-analysis of studies reporting estimates of temporal consistency of dog personality. A thorough literature search identified 31 studies suitable for inclusion in our meta-analysis. Overall, we found evidence to suggest substantial consistency (*r* = 0.43). Furthermore, personality consistency was higher in older dogs, when behavioral assessment intervals were shorter, and when the measurement tool was exactly the same in both assessments. In puppies, aggression and submissiveness were the most consistent dimensions, while responsiveness to training, fearfulness, and sociability were the least consistent dimensions. In adult dogs, there were no dimension-based differences in consistency. There was no difference in personality consistency in dogs tested first as puppies and later as adults (e.g., ‘puppy tests’) versus dogs tested first as puppies and later again as puppies. Finally, there were no differences in consistency between working versus non-working dogs, between behavioral codings versus behavioral ratings, and between aggregate versus single measures. Implications for theory, practice, and future research are discussed.

## Introduction

Personality in humans can be defined as the characteristics of individuals that describe and account for consistent patterns of feeling, thinking, and behaving [1]. Personality in nonhuman animals has often been defined operationally in terms of behaviors that are counted, timed, or rated in standardized tests [2]. In such cases, personality is defined in terms of ‘correlated suites of behavior’, where correlations in behavior can occur across different functional contexts, over time, or some combination of the two [3]–[7]. Personality assessments in species ranging from fish [8], arthropods [9], and cephalopods [10] to birds [11], hyenas [12], and nonhuman primates [13] meet a range of psychometric criteria held as standards for personality assessments in humans [14].

One of the first studies on dog personality was Nobel Laureate Ivan Pavlov's classic work on learning in which he classified dogs into four basic personality types based on their responses to conditioned reflex training [15]. The next significant contribution to personality research in dogs came 50 years later with Scott and Fuller's [16] work on genetic influences on dog behavior; as part of this work these authors attempted to predict later behavior in five different breeds of dogs from behaviors observed earlier in life. Later, in another study of behavioral prediction, Pfaffenberger [17] assessed dog personality using behavioral assessments (i.e., “puppy tests”) to determine which puppies would be best suited for guide-dog work. Since these seminal studies, personality assessment has continued to flourish in the applied working-dog domain [18]–[19] and in studies of the nature and structure of dog personality itself [20]–[22], [23].

Dog personality research has been motivated, in part, by a number of practical concerns. First, there is widespread interest from potential companion dog owners in selecting dogs with personality characteristics that suit their lifestyle. Second, animal shelters and other agencies have an interest in using personality traits to improve the success of the adoption process and to direct care to the animals most in need of attention. Third, agencies focused on reducing the incidence of injuries caused by dog bites have an interest in identifying the individual animals most disposed to aggression. Fourth, working dog practitioners are interested in identifying the individuals with personalities most suited to successful job performance.

All of these cases rest on the assumption that behavior is at least somewhat consistent across time and/or situations. Thus, for example, dog owners try to understand how an adopted dog may behave once it is introduced to their home, or perhaps even months later, based on their observations and interactions with a dog in a shelter or a play area. Similarly, working-dog organizations are interested in identifying puppies as early in life as possible that will grow into adults suitable for working roles. Despite the long history of personality research in dogs and the considerable applied interests, to our knowledge, there are no published quantitative syntheses estimating the strength of consistency of dog personality. Instead, the dog-personality literature is populated with isolated studies in which personality is reported to be sometimes consistent and sometimes not (see Table 1). For example, one study [24] found a strong correlation (*r* = 0.65) between biting/attack scores on two aggression tests held six months apart in adult dogs, and another study [25] found that dog personality assessments conducted as early as 8 weeks of age predicted suitability for police work at 18–24 months of age. Other studies have reported results that were dependent on the particular personality trait that was assessed. For example, one study found that shelter dogs recently adopted by new owners had strongly consistent separation anxiety across two weeks (*r* = 0.71) but did not have strongly consistent fear of unfamiliar dogs (*r*  = 0.10) [26]. Still other studies have reported a lack of consistency for particular traits (e.g., fearfulness), or a decrease in consistency with increasing time intervals between tests [19], [27]. For example, one study reported differences in the consistency of dogs' confidence in semi-novel environments over shorter time intervals (e.g., 2–3 days, *r* = 0.52) versus longer time periods (e.g., 1–3 months, *r* = 0.16) [28]. In short, and as shown in Table 1, there is a lack of consensus about the extent to which personality is temporally consistent in dogs.

**Table 1:** A descriptive summary of the studies used in the meta-analysis.

Author	Year	N	Age (Weeks)	Consistency	No Consistency	Puppy Test	Test Interval (Weeks)	Dog Type	Average Study Effect Size
Beaudet et al.	1994	39	15	1	1	1	9	non-working	0.13
Clark	1995	125	9.5–260	1	0	0	14.5–38.5	non-working	0.44
Goddard and Beilharz	1984	102	12–24	1	1	1	4–39	guide	0.38
Goddard and Beilharz	1985	70	52	1	1	0		guide	0.35
Goddard and Beilharz	1986	102		1	1	1	1-54	guide	0.20
Goleman	2010	144	7.5	1	0	1	44.5		0.83
Hennessy et al.	2001	7–23		1	1	1 & 0	2–24	non-working	0.18
King et al.	2003	12		1	1	0	8.5		0.78
Krauss	1976	76	6–8	1	1	1	2–10		0.16
Ley et al.	2009	50	296	1	0	0	24	non-working	0.88
Maejima et al.	2007	143	72	1	0	0	14	detector	0.31
Martinek and Lat	1969	13	14–208	1	1	1 & 0	42–43		0.47
McPherson	1998	40	3–12	1	1	1	3–9	non-working	0.36
Murphree and Dykman	1965	17	8	1	0	1		non-working	0.53
Netto and Planta	1997	37		1	0	0	24	non-working	0.66
Paroz et al.	2008	30	40	1	1	1		non-working	-0.29
Planta and De Meester	2007	220		1	1	0	52	non-working	0.42
Plutchik	1971	16	208	1	1	0	3–4		0.74
Scott and Bielfelt	1976	239	9.5–12	1	1	1	38–44	guide	0.14
Serpell and Hsu	2001	705–938	24	1	0	1	24	guide	0.53
Sinn et al.	2010	14-498	69–70	1	1	0	0.5–14	military working dog	0.41
Slabbert and Odendaal	1999	167	8–36	1	1	1	48–76	police	0.33
Stephen	2006	10–40		1	0	0	43–4	non-working	0.56
Stephen and Ledger	2007	29–191	157–216	1	1	0	2–6	non-working	0.40
Svartberg	2005	697	72	1	1	0	74	non-working	0.06
Svartberg et al.	2005	40	65–69	1	0	0	4–9	non-working	0.75
Valsecchi et al.	2010	17	56–60	1	1	0	16–48	guide	0.27
Valsecchi et al.	2011	32-34	9.5-260	1	0	0	5.7–21.7	non-working	0.49
Van den Berg et al.	2006	55-70	119.6	1	1	0	224	non-working	0.41
Weiss	2002	75	60	1	1	0	5	non-working	0.27
Weiss and Greenberg	1997	9	68	0	1	0		non-working	0.13
	Studies interpreting their results as indicative of consistency	Studies interpreting their results as indicative of a lack of consistency	Studies interpreting their results as both						
Total	30	21	20						
Percentage	96.8	67.7	64.5						

*Note:* If a range of N was given, two numbers are presented, the smallest number of dogs used from one estimate and the largest number of dogs from another estimate. If authors concluded in their study that they had evidence for consistency for at least one trait, the study was given a ‘1’ for consistency; if authors concluded in their study that they lacked evidence for consistency of any trait, the study was given a ‘0’ for no consistency. Likewise, if authors concluded in their study that they had evidence for a lack of consistency for at least one trait, the study was given a ‘1’ for consistency; if authors concluded that there was at least one trait that was not consistent the study was given a ‘1’ for no consistency. In both cases (‘consistency’ or ‘no consistency’) a zero is given if the study did not report a consistent trait or a trait that was not consistent. A ‘1’ for Puppy test indicates that dogs were tested as puppies (<12 months of age) during the first test period or not (a ‘0’). A ‘1’ and a ‘0’ for Puppy test indicates studies that presented from results from both age categories of dogs. If a range for test interval was given, two numbers are presented, the shortest interval and the largest interval. The average study effect size was calculated by multiplying correlations for each subgroup by the inverse of its variance. The sum of these products was then divided by the sum of the inverses.

These inconsistencies in the literature have led several authors to conclude in qualitative reviews that ‘puppy tests’ are probably not worthwhile [23], [29]–[30]. In addition, there is a lack of consensus regarding the biological and measurement factors that influence personality consistency both in puppies and adult dogs. Here we attempt to quantify temporal consistency of personality in dogs and identify factors that may influence the strength of consistency. We do so by performing a meta-analysis on all known studies of dogs that provide relevant data.

### The Nature of Dog Personality

There is some debate over the number and types of dimensions needed to characterize personality variation in most species of animals [4]. In humans, although not universally accepted, there is now considerable consensus that five broad personality dimensions can capture most of the behavioral variation in people [31]. Some attempts have been made to classify and conceptualize personality in nonhuman animals in terms of a five-dimension model [32]–[33], but there is little consensus about the number or type of personality dimensions that capture most of the observed behavioral variation in dogs.

Several models of dog personality derived from factor-analytic approaches have been proposed. For example, three- [22], [34] and five-dimension models [21] have been used to describe personality variation in companion dogs. One of the most widely used dog personality measures, the Canine Behavioral Assessment and Research Questionnaire (C-BARQ), proposes eleven different personality dimensions in companion dogs [20] and eight different personality dimensions in guide dogs [35]. These C-BARQ dimensions have demonstrated high levels of scale reliability and validity, and the instrument has been used in numerous studies across several populations of dogs [36]–[40].

Taking a different approach, Jones and Gosling [23] used an expert-sorting procedure to classify traits identified in all previous studies of dog personality. The procedure yielded seven broad dimensions (reactivity, fearfulness, activity, sociability, responsiveness to training, submission, aggression, as well as a classification for none/other, which included traits that were not considered to be personality).

These dog personality frameworks all share the goal of attempting to reduce the wide variety of behavior-descriptive terms into a more manageable set of broad dimensions, while also attempting to explain most of the personality variation in dogs. However, as even this brief synopsis illustrates, major differences still exist regarding the number and names of dimensions needed to characterize dog personality. Here we adopted the Jones and Gosling [23] framework, because it is applicable across all dog populations, and many studies examined in the current analyses were already classified in Jones and Gosling's expert-classification [23].

### Potential Factors that Could Affect Personality Estimates

Numerous biological, environmental, and evolutionary influences could contribute to consistency estimates of personality in dogs. Here we focused on two broad areas relevant to temporal consistency in personality: Biological development and measurement methods.

### A. Factors Related to the Biological Development of Dogs

#### Personality dimension

Circulating hormone levels and hormone receptor density have been known to influence behavior [41]–[42]. Moreover, different endocrine systems are thought to have a larger influence on some behaviors than on others. For example, androgens are primary correlates of aggression [43]–[44], and corticosteroids often mediate stress, fear, and risk-taking behaviors [45]–[46].

If different personality dimensions are under different morphological or physiological constraints or if different dimensions undergo different rates of relative fixation throughout ontogeny, then we may expect there to be differences in the consistency estimates across personality dimensions. Personality dimensions that are correlated with developmental systems that are more sensitive to the environment should be less consistent through time than personality dimensions related to more stable developmental systems [47]. Past research does not afford clear predictions regarding which specific dimensions may be more or less consistent, but given the differences in constraints noted above, differences in consistency across different personality dimensions are possible, if not likely.

#### Age at first measurement

In humans, personality tends to be more consistent in adulthood than in adolescence [48]–[50]. Studies of lifetime development of personality are rare in nonhuman animals, but this same pattern of increasing consistency with increasing age has also been observed in a lifetime study of squid [51]. There is some evidence that dogs exhibit more consistency of personality as they grow older [27], and significant personality consistency in adult dogs has been observed over an interval of one to two years [52]. Age-related patterns of personality consistency may result from the energetic or structural costs of changing one's personality, such as changes in neuroendocrine networks [53]. Similarly, increased age may result in increased personality consistency, if individuals choose ecological and social niches appropriate for their personality type, and these environments facilitate and encourage the expression of particular personalities [54]–[55].

#### Working versus non-working dog

Many working-dog programs purpose-breed their own dogs, and dogs designated as breeders in these programs are often chosen based on their physical and behavioral characteristics, the latter of which often includes an individuals' propensity to consistently exhibit appropriate working behaviors. Also, large-scale working-dog programs tend to have standardized processes that could promote similarity that does not occur with non-working dogs [28], [56]. In addition, many of the dogs categorized in the non-working dog category here were sourced from a shelter environment, which is probably unstable and stressful [57] relative to rearing environments experienced by working dogs. Based on these observations, we expected working dogs to have higher estimates of consistent personality relative to non-working dogs.

### B. Factors Related to Personality Measurements

Some factors that influence estimates of personality consistency may be related to the testing instrument; these factors could affect consistency estimates even if the actual underlying personality was stable. We focus on factors known to affect measurement in previous studies of human and nonhuman personality.

#### Interval between measurements

Environmental influences such as changes in the social and developmental environment are more likely to occur during longer (versus shorter) intervals between tests. Some dog studies support the idea that shorter between-test intervals yield higher estimates of consistency than longer intervals [28].

#### Behavioral codings versus behavioral ratings

At least two different types of methods can be used when measuring personality: behavioral codings and behavioral ratings [3], [58]. Behavioral codings typically attempt to measure observed, discrete classifications of behavior, often generated from an ethogram, such as the frequency and duration of a particular posture. For example, one study used the number of lines (marked on the floor of a test arena) that a dog crossed as an indicator of locomotor activity [59]. Ratings typically consist of broader judgments regarding a dog's standing on a behavioral trait made by people familiar with the dog. For example, in one study dogs were rated on a 1–5 Likert scale according to their playfulness with a rag in a standardized test [34].

In some instances, the reliability of behavioral ratings may be lower than that of behavioral codings because a rater has a restricted relationship to the target subject, such as in cases where raters only perform veterinary or feeding duties [60]. In other instances, empirical comparisons of the reliability of behavioral ratings versus behavioral codings of the same animals have shown behavioral codings to be less reliable than behavioral ratings [56]. This advantage of behavioral ratings can be attributed to the fact that behavioral ratings tend to reduce error variance by accounting for situational effects and incorporating longer behavioral trends. These factors would suggest that dog personality consistency estimates will be higher when behavioral ratings rather than behavioral codings are used.

#### Single versus aggregate measures

Psychometric principles suggest that aggregate measures (i.e., sum or average of multiple observed behaviors) will tend to be more reliable than single measures because the random, nonsystematic error in the different multiple measures will tend to cancel each other out. In the human domain, aggregated measures yield greater consistency than do single measures [61]–[63]. In dogs, aggregate measures have been shown to be more powerful predictors than single measures with regards to military working-dog certification outcomes [28].

#### Similarity of tests

In many studies, two different test methods are used to measure the same personality trait. For example, at one time point, behavior may be measured using a standardized test situation and behavioral coding (e.g., shelter tests for behavior) but at a second time, a rating form might be used [64]. Studies that used the same test to measure a certain personality dimension are likely to yield higher consistency estimates than studies that used different tests across time, due to reduced method variance in the former [65].

#### Summary

A great deal of research has accumulated on the temporal consistency of dog personality. Here we use meta-analytic methods to quantitatively summarize the overall consistency of dog personality and the factors that influence it. In line with our synopsis above, we expected our meta-analysis to reveal that dog personality was moderately consistent over time, but the absolute level of consistency may vary depending on the personality dimension being assessed. Further, we predicted that personality will likely be more consistent when dogs are tested as adults, when dogs are sourced from working dog programs, when behavioral ratings are used, when aggregate measures are used, when shorter test intervals are used, and when the same test is administered across test occasions.

## Methods

### Literature Search Procedures

To identify as many relevant studies as possible, we first searched *PsychInfo, Biosis, Web of Science*, and *ProQuest Dissertation and Theses* electronic databases for documents catalogued before August 2011. In each database, 12 keyword searches were performed, derived from all combinations of a 6 (temperament test, personality test, behavior evaluation, prediction, temperament, personality) x 2 (dog, puppy) matrix. Next, we examined the reference sections of all of articles obtained through the database searches to determine if any cited works had titles that also might be relevant to the topic. Also, we conducted a Social Science Citation Index search on two previous studies that were heavily cited in the topic of dog personality [18], [23]. Finally, to reduce bias potentially introduced by our limited perspective we also asked eight dog-personality experts if they knew of any studies we had missed.

### Inclusion Criteria

For a study to be included in the meta-analysis, several criteria had to be met. First, studies had to have tested the consistency of dog behavior across two or more time points. Second, studies had to provide a bivariate correlation (*r*) of this relationship or provide sufficient information to compute or convert the estimates to an *r-*value. Third, to ensure that all estimates met a threshold of peer-reviewed or committee-reviewed scholarship standards, studies had to be either a published report or a completed dissertation or thesis. Finally, to ensure that the estimates were assessing repeated measurements of the same construct, the second test assessment method had to be at least conceptually similar (i.e., measure the same dimension) as the first.

Our search yielded 107 studies. Each was examined by the first or second author and 79 studies were excluded based on our selection criteria. The most common causes of exclusion were because the studies presented data in a way that was impossible to convert to a bivariate correlation [66] or because the test two measurements did not clearly measure the same dimension at two different time points [67]. In 19 cases where the article did not report sufficient information to compute *r*, but data from the study was relevant and recent (i.e., published in 2000 or later), we emailed the study's authors to request the required information. Five author groups responded, and three were able to provide the additional relevant data. In total, 31 studies (28 published studies and 3 unpublished dissertations) with a total of 822 estimates of consistency were included in the meta-analysis.

### Information Coded from Studies

For each study, we recorded information regarding authors, year, title, journal, source we found each study from, the number of subjects, the age of the subjects at the first test in weeks (if more than one age was given for a single estimate, we used the average age), the type of dogs (working or non-working dog), the name of the trait(s) given from the study, a description of the test domains (e.g., response to novelty), the average interval between tests (in weeks), the test methods (codings or ratings), if the measures of personality reflected a single measure or an aggregate set of measures, and if test one was exactly the same or only conceptually the same as test two.

The studies amassed here reported 213 unique trait names, which precluded any comparison of consistency estimates at the trait level due to a lack of sufficient statistical degrees of freedom. Therefore, to compare traits assessed in the studies, we classified all trait names given in each study in terms of the personality dimensions described in the Jones and Gosling [23] seven-dimension framework (see Table 2). For studies that were not included in the Jones and Gosling [23] review, we used descriptions of trait names, test domains, and test procedures given by authors to match them to traits already classified by Jones and Gosling [23] (see Table 3). Jones and Gosling [23] questioned the wisdom of separating the fearfulness and reactivity dimensions and only one study in our review provided estimates that could potentially be classified exclusively as ‘reactivity’ [68] so we combined these two dimensions, using the label ‘fearfulness.’ We also excluded descriptions of traits considered as ‘other’, because Jones and Gosling [23] considered these traits as not being related to dog personality. In cases where traits from studies were identified as straddling more than one of the seven dimensions, we assigned the trait to both categories (see Table 3).

**Table 2:** Description of the Jones & Gosling 2005 personality framework used for meta-analysis.

	Dimension Description
Activity	Often assessed by placing a puppy or dog in an empty arena with gridlines on the floor and seeing how many times the puppy or dog crosses the lines. Includes traits labeled as ‘activity’, ‘locomotor activity’, and ‘general activity’.
Aggression	Indexed by behaviors such as biting, growling, and snapping at people or other dogs. Often assessed through having strangers approach the dog in a threatening manner. Includes traits labeled as ‘stranger directed fear or aggression’, ‘owner-directed aggression’, ‘dog-directed fear or aggression’, ‘sharpness’, and the ‘willingness to bite a human being’.
Sociability	Indexed by such behaviors as initiating friendly interactions with people and other dogs. Primarily assessed in meetings between dogs and an unfamiliar person. Includes traits labeled ‘extraversion’, ‘affection demand’, and ‘affability’.
Responsiveness to training	Indexed by such behaviors as working with people, learning quickly in new situations, playfulness, and overall reaction to the environment. Related to a dog's tendency to stay focused and engaged in a given activity. Normally assessed through giving dogs puzzles to solve, willingness to work with a person, and retrieval tests. Includes traits labeled ‘distractability’, ‘focus’, ‘problem solving’, ‘willingness to work’, and ‘cooperative’.
Submissiveness	The opposite of dominance. Dominance can be judged by observing which dogs bully others, and which guard food areas and feed first. Submission can also be reflected by such behaviors as urination upon greeting people.
Fearfulness (with Reactivity)	Exhibited by signs of excitement, pacing or running around, avoidance of novel stimuli, and barking. Shaking and a tendency to avoid novel stimuli without approaching them. Includes trait labels ‘courage’, ‘confidence’, ‘self-confidence’, ‘apprehension’, ‘dog-directed fear or aggression’, and ‘timidity’. Indexed by such behaviors as repeated approach/ avoidance of novel objects, raised hackles, and increased activity in novel situations. Assessed through procedures such as presenting a novel object or series of novel objects to a puppy and recording its subsequent behavior. Includes traits labeled as ‘excitability’, ‘sound reaction’, and ‘heart reactivity’.

**Table 3:** Classification of study traits into the Jones and Gosling (2005) 7-dimension dog personality framework.

Author	Study trait name	Jones and Gosling (2005) dimension classification
Beadet et al. (2003)	Number of movements	Activity
	Social tendencies	Submissiveness
Clark (1995)	Dominance	Submissiveness
Goddard & Beilharz (1984)	Avoid experimenter	Fearfulness
	Ears erect	Fearfulness
	Fear on walk	Fearfulness
	Jumping behind dog	Fearfulness
	Party whistle	Fearfulness
	Pistol fired	Fearfulness
	Rapid head movements	Fearfulness
	Toy horse reaction	Fearfulness
	Aggression-dominance	Multiple
	Confidence	Fearfulness
	Activity	Activity
	Avoids	Fearfulness
	Balks	Fearfulness
	Come	Responsiveness to training
	Fear of crumbled paper	Fearfulness
	Fear of dog	Fearfulness
	Fear of gunshot	Fearfulness
	Fear of ice cream container	Fearfulness
	Fear of objects	Fearfulness
	Fear of party whistle	Fearfulness
	Fear of rubber ball	Fearfulness
	Fear of steps	Fearfulness
	Fear of surfboard	Fearfulness
	Fear of toy car	Fearfulness
	Fear on walk	Fearfulness
	Fetch	Responsiveness to training
	Puppy test index	Multiple
	Sit	Responsiveness to training
Goleman (2010)	Puppy test outcome	Other
	Sociability (tester)	Sociability
Hennessy et al. (2001)	Flight	Fearfulness
	Locomotor activity	Activity
	Sociability	Sociability
	Solicitation	Other
	Timidity	Fearfulness
	Wariness	Fearfulness
King et al. (2003)	Heart rate readings	Fearfulness
	Maximum withdrawals	Fearfulness
	Novel object approach	Fearfulness
	Number of entries (closed arms)	Activity
	Number of entries (lit)	Activity
	Number of entries (open arms)	Activity
	Number of entries (toy)	Fearfulness
	Time in lit compartment	Other
Krauss (1976)	Activity level	Activity
	Balks	Responsiveness to training
	Commands	Responsiveness to training
	Curiosity	Fearfulness
	Elevation dominance	Multiple
	Fear of novel stimuli	Fearfulness
	Fear of strangers	Multiple
	Fetch	Responsiveness to training
	Following	Sociability
	Noise sensitivity	Fearfulness
	Person distraction	Responsiveness to training
	Pulls	Responsiveness to training
	Puppy healing	Responsiveness to training
	Quieting	Responsiveness to training
	Restraint dominance	Multiple
	Sit	Responsiveness to training
	Social attraction	Sociability
	Social dominance	Submissiveness, aggression
	Whistle	Fearfulness
Ley et al. (2009)	Amicability	Sociability
	Extraversion	Activity
	Motivation	Responsiveness to training
	Neuroticism	Fearfulness
	Training focus	Responsiveness to training
Maejima et al. (2007)	Desire for work	Multiple
	Distractibility	Multiple
Martinek & Lat (1969)	Grid crossings	Activity
	Movement	Activity
	Movement and rearing	Activity
	Rearing	Activity
	Sniffing	Activity
	Sum of grid crossings	Activity
	Vocalization	Fearfulness
McPherson (1998)	Absence of familiar person (barking)	Fearfulness
	Absence of familiar person (sit at door)	Fearfulness
	Absence of familiar person (vocalize)	Multiple
	Absence of familiar person (whining)	Fearfulness
	Approach familiar person	Sociability
	Approach stranger	Multiple
	Approach stranger (latency)	Multiple
	Barking during separation	Fearfulness
	Contacting exit	Fearfulness
	Cringe at familiar person	Multiple
	Cringe at stranger	Multiple
	Interaction with familiar person	Multiple
	Interaction with stranger	Multiple
	Interaction with stranger, absent of familiar person	Multiple
	Investigate familiar person	Multiple
	Investigate stranger	Multiple
	Jump on familiar person	Multiple
	Jump on stranger	Multiple
	Look at familiar person	Sociability
	Look at stranger	Multiple
	Rollover for stranger	Submissiveness
	Vocalize during separation	Sociability
	Whining during separation	Fearfulness
Murphree & Dykman (1965)	Exploratory activity	Activity
Netto & Planta (1997)	Attack behavior	Aggression
	Biting/attack	Aggression
	Snapping	Aggression
Paroz et al. (2008)	Play (handler)	Responsiveness to training
	Play (stranger)	Responsiveness to training
	Relationship with handler	Multiple
	Self confidence	Fearfulness
	Temperament	Other
Planta & De Meester (2007)	Aggression	Multiple
Plutchik (1971)	Activity	Activity
	Approach	Fearfulness
	Avoidance	Fearfulness
	Contact time (object)	Fearfulness
	Enter area latency	Fearfulness
	Non-responses	Other
	Urinations	Other
Scott & Bielfelt (1976)	Body sensitivity	Other
	Closeness	Other
	Come	Responsiveness to training
	Ear sensitivity	Other
	Fetch	Responsiveness to training
	Footing crossing	Fearfulness
	Heel	Other
	Sit	Responsiveness to training
	Success	Other
	Traffic	Fearfulness
	Trained response	Responsiveness to training
	Willing in training	Responsiveness to training
Serpell & Hsu (2001)	Attachment	Sociability
	Chasing	Multiple
	Dog directed fear/aggression	Multiple
	Energy level	Activity
	Nonsocial fear	Fearfulness
	Owner directed aggression	Aggression
	Stranger directed fear/aggression	Multiple
	Trainability	Responsiveness to training
	Trainability	Responsiveness to training
Sinn et al. (2010)	Attention transfer	Responsiveness to training
	Defense	Multiple
	Environmental sureness	Multiple
	Frontal bite	Aggression
	Gun sureness	Fearfulness
	Human focus	Multiple
	Non-threat bite quality	Aggression
	Object focus	Multiple
	Physical possession	Other
	Possession	Responsiveness to training
	Pursuit bite	Aggression
	Search activity	Activity
	Search focus	Activity
	Search stamina	Activity
	Sharpness	Multiple
	Static object interest	Multiple
	Threat bite quality	Aggression
	Threat defense	Multiple
	Thrown object interest	Responsiveness to training
Slabbert & Odendaal (1999)	Aggression	Aggression
	Gun	Fearfulness
	Obstacle	Responsiveness to training
	Retrieve	Multiple
	Startle	Fearfulness
Stephen & Ledger (2007)	Aggression of person reaching out	Aggression
	Aggression of unfamiliar dogs	Aggression
	Aggression of unfamiliar people	Aggression
	Aggression of veterinarian	Aggression
	Anxiety at the vet	Multiple
	Anxiety when alone	Fearfulness
	Attentiveness come	Responsiveness to training
	Attentiveness sit	Responsiveness to training
	Attentiveness stay	Responsiveness to training
	Chewing furniture	Other
	Excessive vocalization	Other
	Excitement of familiar people	Multiple
	Excitement of unfamiliar people	Multiple
	Fear of loud noises	Fearfulness
	Fear of unfamiliar dogs	Multiple
	Fear of unfamiliar people	Multiple
	Fear of veterinarian	Multiple
	Sexual mounting	Other
	Stealing food	Other
Stephen (2006)	Anxiousness	Fearfulness
	Distractibility	Responsiveness to training
	Excitability	Multiple
	Exploration	Multiple
	Fearfulness	Fearfulness
	Playfulness	Multiple
Svartberg (2005)	Aggression (distance play)	Aggression
	Aggression (ghosts)	Aggression
	Aggression (sudden appearance)	Aggression
	Aggressiveness	Aggression
	Chase proneness	Multiple
	Curiosity/fearfulness	Fearfulness
	Distance playfulness	Sociability
	Playfulness	Sociability
	Sociability	Sociability
	Aggression	Aggression
	Boldness	Multiple
	Chase-proneness	Aggression
	Curiosity/fearlessness	Fearfulness
	Playfulness	Sociability
	Sociability	Sociability
Valsecchi et al. (2010)	Approach puppet	Fearfulness
	Contact with human	Sociability
	Contact with owner	Sociability
	Contact with stranger	Sociability
	Drink	Other
	Exploration	Activity
	Exploration (puppet)	Fearfulness
	Greeting owner	Sociability
	Greeting stranger	Sociability
	Locomotion	Activity
	Orientation to door	Other
	Passive	Fearfulness
	Playfulness	Other
	Proximity seeking	Multiple
	Puppet fear	Fearfulness
	Scratch the door	Other
Valsecchi et al. (2011)	Handling behavior	Sociability
	Temperament	Other
van den Berg et al. (2006)	Dog directed aggression	Multiple
	Stranger directed aggression	Aggression
Weiss & Greenberg (1997)	Attention/distraction	Multiple
	Dominance	Submissiveness
	Excitement	Fearfulness
	Fear/submission	Multiple
	Performance	Other
Weiss (2002)	Activity level	Activity
	Activity vertical	Activity
	Aggregate	Other
	Fetch	Responsiveness to training
	Jumping on tester	Activity
	Other dog	Multiple
	Pinch	Other
	Pinch	Other
	Potential	Other
	Sensitivity	Other
	Sound sensitivity	Fearfulness
	Success	Other
	Walk	Responsiveness to training

*Note:* ‘Multiple’ means we could not classify the study trait name in to a single dimension, so the trait composed of multiple dimensions. The trait estimate was not used in calculating the personality dimension moderator because of this.

We recorded several additional characteristics, including the analysis strategy that authors used to determine the relationship between tests (e.g., correlation), the result type (e.g., odds ratio or Pearson’s *r*, etc.), the direction of the effect, and the value of *r*. In studies that reported results from logistic regression, we converted log odds ratios to Cohen’s *d*, and then converted *d* to *r* [69]. For studies that reported only a *p*-value, we converted *p* to its associated one-tailed standard *z* and then converted *z* to *r* [70]. If a study reported a test-retest reliability coefficient based on opposite response scales across tests (e.g., higher numbers indicated greater presence of a trait on the response scale for the first test while higher numbers indicated lower presence of the trait on the response scale for the second test), we re-keyed the coefficient.

### Data Reliability

The second author recorded information for all studies, and the first author independently recorded data from 10 of the studies to assess reliability. There was 96% agreement between the two authors for those 10 studies across 22 variables. The few disagreements were about how to classify the original personality trait name from the study into the Jones and Gosling [23] framework; so for personality dimension classifications, the first author also categorized trait names from all studies according to the Jones and Gosling [23] framework. Discrepancies were noted and discussed, and agreement was reached in all cases.

### Methods of Data Integration

Before conducting any analyses, we examined the distribution of effect sizes to determine if our dataset contained statistical outliers. Grubbs’ test was applied [71]–[72] and no outliers were identified.

We employed Duval and Tweedie’s [73]–[74] trim-and-fill procedure to test whether the distribution of effect sizes used in the analyses was consistent with that expected if the estimates were normally distributed. If the distribution of observed effect sizes was skewed, indicating a possible bias created either by the study retrieval procedures or by data censoring on the part of authors, the trim-and-fill method provides a way to estimate the values from missing studies that need to be present to approximate a normal distribution. The procedure then imputes these missing values, permitting an examination of an estimate of the impact of data censoring on the observed distribution of effect sizes.

#### Fixed and random error

There are two common ways to conceptualize meta-analysis: fixed effects and random effects models. These models differ in their theoretical assumptions and also how mean effect sizes and significance are calculated [75]. Fixed error models assume that studies in a meta-analysis are sampled from a single population with a fixed` average effect size. Random effect models assume that the average effect size varies randomly from study to study: studies in a meta-analysis come from multiple populations that have different average effect sizes, so study/population effect sizes can be thought of as being sampled from a ‘superpopulation’ [76]. One consequence of these assumptions lies in the statistical calculation of error. Fixed error models assume error is introduced because of sampling studies from a population of studies. This error is also assumed in random effects models, but in addition there is error created by sampling the populations from a superpopulation [77]. Fixed effect models are common but there is considerable theoretical and empirical evidence that real-world data likely fit random effects models more closely [77]–[78]. For this reason, we reported average effect sizes computed under the random effects model, but conducted our analyses using the fixed effect model too. In fact, random effects are computed in an iterative fashion based on fixed effects. We report within-class goodness-of-fit values (*Q_w_*) using fixed-effect weights to assess the between-studies dispersion and tests of homogeneity for all studies within a group. *Q_w_* values computed using random-effects weights would not be appropriate for this purpose. Average effects computed using fixed-effect weights and other fixed effect model results are presented in the supporting information.

#### Calculating average effect sizes

A weighting procedure was used to calculate average effect sizes, both within and across studies. Each independent correlation was first multiplied by the inverse of its variance. Then, the sum of these products was divided by the sum of the inverses [79]. This weighting procedure is generally preferred because it gives greater weight to effect sizes based on larger samples since larger samples give more precise population estimates. Also, 95% confidence intervals were calculated for the overall weighted average effect. If the confidence interval did not contain zero, then the null hypothesis that there is no consistency in dog personality across time was rejected.

#### Identifying independent hypothesis tests

One problem that arises in calculating effect sizes involves deciding what constitutes an independent estimate of effect. Here, we used a shifting unit of analysis approach [80]. In this procedure, each effect size associated with a study is first used as if it were an independent estimate of the relationship. For example, if a single study provided correlations across two time points for both aggression and fearfulness, two separate correlations were recorded. However, for estimating overall consistency in dog personality, these two correlations were averaged prior to analysis, so that the study only contributed one effect size. To calculate the overall weighted correlation and confidence interval, this one effect size would be weighted by the inverse of its variance and sample size. However, in an analysis that examined consistency in aggression and fearfulness separately, the study was permitted to contribute one effect size to each mean effect size. The shifting unit of analysis approach retains as much data as possible from each study while holding to a minimum any violations of the assumption that data points are independent.

#### Tests for moderators of effects

Possible moderators of the consistency of dog personality between two time points were tested using homogeneity analyses and meta-regression techniques [79]–[81]. Homogeneity analyses compare the amount of variance in an observed set of effect sizes with the amount of variance that would be expected by sampling error alone. The analyses can be carried out to determine whether the variance in a group of individual effect sizes varies more than predicted by sampling error. Within a fixed effects model, the homogeneity of the set of effect sizes is tested using a within-class goodness-of-fit statistic (*Q_w_*), which has an approximate chi-square distribution with *k* – 1 degrees of freedom, where *k* equals the number of effect sizes. Thus, a significant *Q_w_* statistic would indicate systematic variation among effect sizes and suggest that moderator variables be examined or that a random effects model may be most appropriate for the data [80]. Homogeneity analyses can also be used to determine whether multiple groups of average effect sizes vary more than predicted by sampling error alone. In this case, statistical differences among groups of estimates are tested by computing the between-class goodness-of-fit statistic (*Q_b_*), which has a chi-square distribution with *p* – 1 degrees of freedom, where *p* equals the number of groups. A significant *Q_b_* statistic indicates average effect sizes vary between categories of the moderator variable more than predicted by sampling error alone. This strategy is analogous to testing for group mean differences in an analysis of variance.

Meta-regression techniques were used when moderators were continuous (i.e., interval between tests measured in weeks). Two values assess the fit of a weighted regression model. First, the *Q* regression (*Q*_r_) examines the total variability associated with the predictors in the regression model. *Q*_r_ has *p* degrees of freedom, where *p* equals the number of predictors. A significant *Q*_r_ indicates the regression model explains significant variability in effect sizes and that at least one regression coefficient is significantly different from zero. The weighted sum-of-squares residual (*Q*_e_) examines the variability unaccounted for by the model. *Q*_e_ has *k* –*p* – 1 degrees of freedom, where *k* represents the number of effect sizes and *p* equals the number of predictors. A significant *Q*_e_ indicates that after removing variability based on the predictor values, the effect sizes remain heterogeneous [79]. While in a fixed effect model, a significant *Q*_e_ may suggest that a random effects model may be more appropriate for data, in a random effects model, this residual heterogeneity is assumed to be composed entirely of sampling error and will generally be small [79]. Meta-regression is analogous to testing for effects of a set of predictors on an outcome variable in a multiple regression model. For this study, we performed an unrestricted maximum likelihood (ML) random effects regression, using the consistency correlation as the dependent variable.

All analyses were conducted using Comprehensive Meta-Analysis statistical software package Version 2 [82].

## Results

Table 1 summarizes descriptive information from the 31 studies included in our analyses. 96.8% of the studies reported significant consistency in at least one trait, 67.7% of studies reported a null effect in at least one trait, and 64.5% of studies reported both a significant and non-significant effect in at least one trait. The sample size of each study varied considerably, ranging from 7 dogs [59] to 938 dogs [35], with an average of 84 dogs per study. The time interval between measures across studies varied as well, ranging from 3 days [83] to 224 weeks [37], with an average interval of 21 weeks. The age at which a dog was first tested also varied considerably across studies, ranging from 3 weeks of age [84] to 296 weeks of age [85], with a mean of 49 weeks of age. 65% of dogs tested were non-working dogs. For working dog studies, 6 studies surveyed guide dogs, 1 study surveyed military working dogs, 1 study surveyed police dogs, and 1 study surveyed detector dogs. Four studies did not specify what type of dog (working or non-working) was being tested. A majority (72%) of studies used behavioral ratings at both test periods, but of these, a majority (76%) of the raters did not own or care for the dog. More studies (59%) used aggregate rather than single measures at both test periods. More studies (57%) used the exact same measure at both time points than used conceptually similar, but different measures.

### Overall Consistency in Dog Personality

Of the 822 effect sizes, 708 were in a positive direction, and 114 were in a negative direction. The effect sizes for estimates of consistency for single traits ranged from *r = *−0.73 [40] to *r*  = 1.00 [86]. The overall effect size was moderate and significantly greater than zero (*k* = 31, *r* = 0.43, 95% CI  = 0.35, 0.50). Additionally, the tests of the distribution of effect sizes revealed that we could reject the hypothesis that the effects were estimating the same underlying population value (fixed effects: *Q*_30_  = 3393.30, *p*<0.001), suggesting that we could proceed with moderator analyses and that a random effects model is likely most appropriate. Trim-and-fill analyses using random error models, explicitly searching for possible missing effects on the left side of the distribution (those that would reduce the size of the positive average *r*) revealed no evidence for possible data censoring.

### Moderators of Consistency Estimates

#### Personality dimension

Personality traits that could not be clearly classified into a single Jones and Gosling [23] dimension were not used in the moderator test (i.e., all estimates used fell into only one dimension). Using the six dimensions from Jones and Gosling’s [23] framework and including dogs across all ages, the average weighted consistency correlation across time points did not vary significantly by dimension (*Q*_5_  = 6.60, *p* = 0.25). Consistency estimates for all personality dimensions were significantly different from zero, ranging from 0.28 for responsiveness to training to 0.50 for aggression (Table 4).

**Table 4:** Results of moderator analysis.

				95% Confidence Interval	95% Confidence Interval	
*Moderators*		*k*	*r*	*Low Estimate*	*High Estimate*	*Q-within*
**Personality Dimension**						2531.97^**^
Activity		12	0.36^**^	0.25	0.46	162.75^**^
Aggression		8	0.50^**^	0.30	0.65	642.17^**^
Fearfulness		22	0.39^**^	0.30	0.48	829.30^**^
Responsiveness to Training		11	0.28^**^	0.15	0.39	346.70^**^
Sociability		9	0.47^**^	0.22	0.66	548.07^**^
Submissiveness		4	0.42^**^	0.36	0.48	2.98
**Age at First Measurement**						3315.33^**^
Puppy		14	0.30^**^	0.19	0.41	3315.33^**^
Puppy/Puppy		8	0.38^**^	0.26	0.49	738.04^**^
Puppy/Adult		5	0.40^**^	0.14	0.60	527.30^**^
Adult		17	0.51^**^	0.39	0.61	1761.47^**^
**Working Versus Non-Working Dog**						2700.27^**^
Non-Working		16	0.41^**^	0.27	0.53	1963.18^**^
Working		10	0.36^**^	0.26	0.46	737.27^**^
**Behavioral Codings Versus Behavioral Ratings**						2977.75^**^
Codings		8	0.42^**^	0.29	0.53	294.84^**^
Ratings		21	0.42^**^	0.32	0.51	2682.91^**^
**Single Versus Aggregate Measures**						2299.05^**^
Single		20	0.45^**^	0.32	0.56	394.85^**^
Aggregate		16	0.40^**^	0.33	0.46	1904.20^**^
**Similarity of Tests**						2876.06^**^
Same		22	0.49^**^	0.42	0.56	1979.39^**^
Different		16	0.27^**^	0.16	0.37	896.67^**^

+ p<0.10, ^*^P<0.05, ^**^p<0.01, k =  number of studies, r =  bivariate correlation, Q-within was calculated from fixed-effects models.

#### Age at first measurement

The association between the age of the dog at first test and the personality consistency estimate was assessed by categorizing estimates according to whether dogs were puppies (<12 months old) or adults (>12 months old) at the first test. For both age categories, consistency estimates were significantly different from zero and significantly different from each other (*Q*_1_ = 6.58, *p* = 0.01; Table 4). Of note, the average weighted adult personality consistency estimate (*r* = 0.51) was 1.7 times as large as the puppy personality consistency estimate (*r* = 0.30).

To test the effectiveness of ‘puppy tests’ more explicitly we also examined whether consistency estimates were different between puppies first tested as puppies and then tested again as puppies (average interval between tests  = 7.84 weeks) versus puppies first tested as puppies and then tested as adults (i.e., average interval  = 47.52 weeks). For both categories, consistency estimates were significantly different from zero (*r* = 0.38 and 0.40, respectively), but were not different from one another (*Q*_1_ = 0.02, *p* = 0.90).

Next, we examined whether consistency varied by personality dimension separately for puppies and adult dogs (Table 5). Among puppies, the estimates for all dimensions except for sociability were significantly different from zero, ranging from 0.16 for responsiveness to training to 0.51 for aggression. Further, this variability in consistency by personality dimensions was significantly greater than would be expected by sampling error alone (*Q*_5_ = 22.12, *p*<0.001). A series of pair-wise comparisons suggested the largest effect sizes for consistency in puppies were for aggression (*r* = 0.51) and submissiveness (*r* = 0.43), which were not significantly different from each other. Fearfulness (*r* = 0.24) and responsiveness to training (*r* = 0.16) were the least consistent dimensions and were not significantly different from each other. Responsiveness to training and fearfulness were significantly less consistent than aggression and submissiveness but not activity. Activity (*r* = 0.26) was significantly less consistent than submissiveness and marginally less significant than aggression (Table 6).

**Table 5:** Interaction of personality dimensions and age of dog.

			95% Confidence Interval	95% Confidence Interval	
	*k*	*r*	*Low Estimate*	*High Estimate*	*Q-within*
**Puppy**					254.63^**^
Activity	7	0.26^**^	0.10	0.40	88.29^**^
Aggression	2	0.51^**^	0.28	0.68	19.31^**^
Fearfulness	10	0.24^**^	0.12	0.36	344.04^**^
Responsiveness to Training	6	0.16^**^	0.04	0.28	112.47^**^
Sociability	4	0.42^*^	−0.06	0.75	373.20^**^
Submissiveness	3	0.43^**^	0.36	0.49	0.97
**Adult**					1273.14^**^
Activity	7	0.50^**^	0.30	0.66	82.69^**^
Aggression	6	0.49^**^	0.27	0.67	431.87^**^
Fearfulness	13	0.51^**^	0.31	0.66	430.95^**^
Responsiveness to Training	4	0.48^**^	0.15	0.72	214.85^**^
Sociability	6	0.47^**^	0.17	0.69	224.48^**^
Submissiveness	1	−0.13	−0.73	0.59	0.00

+ p<0.10, ^*^p<0.05, ^**^p<0.01, k =  number of studies, r =  bivariate correlation, Q-within was calculated from fixed-effects models.

**Table 6:** Contrasts (Q-values) for personality dimensions in puppies.

		Aggression	Fearfulness	Responsiveness to Training	Sociability	Submissiveness
Puppy						
	Activity	3.36+	0.02	0.92	0.47	4.58^*^
	Aggression		4.06^*^	6.64^*^	0.14	0.51
	Fearfulness			0.89	0.57	7.53^**^
	Responsiveness to Training				1.15	15.55^**^
	Sociability					0.00

+ *p*<0.10, *p<0.05, ** *p*<0.01, all values are Q values.

In contrast, there was no significant variation in consistency by personality dimension among adult dogs, *Q*_5_  = 2.70, *p* = 0.75 (Table 4). Rather, with the exception of a non-significant consistency estimate for submissiveness (*r* = −0.13), consistency estimates among dogs for all other personality dimensions were significantly different from zero and were fairly similar, ranging between 0.47 for sociability to 0.51 for fearfulness. It should also be noted that the estimate for the submissiveness dimension among adult dogs was based on only a single study and should be interpreted with caution.

#### Working versus non-working dogs

Consistency estimates for both working and non-working dogs were significantly different from zero (*r* = 0.36 and 0.41, respectively), but there was no difference in consistency of dog personality between the two groups (*Q*_1_ = 0.29, *p* = 0.59; Table 4).

#### Interval between measurements

To evaluate the association between test interval and consistency, we used the test interval (in weeks) as a continuous variable in an unrestricted ML meta-regression. The model was significant (*Q*_1_  = 13.57, *p*<0.001), with ∼2% of the variability in consistency correlations accounted for by test interval. As the test interval increased, the magnitude of consistency decreased (Table 7).

**Table 7:** Unrestricted ML meta-regression for ‘time interval between tests’ moderator.

*Variable*	*B*	*p*
Interval	−0.002	<0.001
Regression constant	0.39	
Overall model	Q(1) = 13.57	
Residual	Q(786) = 794.30	
Total	Q(787) = 807.87	
R^2^ = 0.02		

To further investigate the effectiveness of ‘puppy tests’, we examined the association between test interval and age of dog at first test on the overall effect size of consistency. We separated test interval into short (<10 weeks), medium (10–24 weeks), and long (>24 weeks) categories and used the previous designations for puppies (<12 months old) and adults (>12 months). For puppies, estimates for all interval categories were greater than zero and there was no difference between length-of-interval category (*Q*_2_  = 1.586, *p* = 0.45). For adults, all estimates for interval categories were greater than zero and there was a marginal statistical difference between interval categories (*Q*_2_ = 4.46, *p* = 0.096). Short intervals (*r* = 0.60) tended to result in higher consistency estimates than long intervals (*r* = 0.32; *Q*_1_ = 4.69, *p* = 0.03). There was no difference between short and medium intervals (*Q*_1_ = 0.25, *p* = 0.62), or between medium and long intervals (*Q*_1_ = 1.38, *p* = 0.24; Table 8).

**Table 8:** Interaction of test interval and age of dog.

			95% Confidence Interval		
	*k*	*r*	*Low Estimate*	*High Estimate*	*Q-within*
**Puppy**					477.54^**^
Short	6	0.25^**^	0.12	0.38	284.98^**^
Medium	5	0.34^**^	0.19	0.48	162.25^**^
Long	7	0.39^**^	0.19	0.56	524.93^**^
**Adult**					1272.26^**^
Short	8	0.60^**^	0.49	0.70	182.60^**^
Medium	7	0.53^**^	0.25	0.73	337.74^**^
Long	4	0.32^*^	0.06	0.55	139.30^**^

+ p<0.10, ^*^p<0.05, ^**^p<0.01, k =  number of studies, r =  bivariate correlation.

*Note:* Short intervals were those where both behavioral assessments were conducted less than 10 weeks of one another; medium intervals had test intervals of 10 to 24 weeks, and long test intervals were greater than 24 weeks apart.

#### Behavioral codings versus behavioral ratings

The consistency estimates for both behavioral codings (*r* = 0.42) and ratings (*r* = 0.42) were significantly different from zero, but the two estimates were not significantly different from one another (*Q*_1_ = 0.00, *p* = 0.99; Table 4). Note that for this moderator test we used only studies that used the same method of measurement on both test occasions (e.g., behavioral codings or ratings at both test 1 and test 2, but not a combination of the two methods across time).

#### Single versus aggregate measures

Consistency estimates for aggregate trait measures (*r* = 0.45) and single trait measures (*r* = 0.40) were significantly different from zero, but not different from one another (*Q*_1_ = 0.55, *p* = 0.46; Table 4). Note that for this moderator test we used only studies that used the same measure for both tests (e.g., single measures or aggregate measures at both test 1 and test 2).

#### Similarity of assessments

There was a significant difference in consistency of dog personality when the exact same test was given across time points compared to when the test was methodologically different (*Q*_1_ = 11.72, *p* = 0.001; Table 4). Consistency was 1.8 times greater when the tests across the two measurement time points were identical (*r* = 0.49) compared to when different tests were given (*r* = 0.27).

## Discussion

The concept of personality implies that behavior shows some level of temporal consistency. In dogs, personality consistency is especially relevant because success in most tasks depends on a dog's ability to express predictable and appropriate behaviors. Canine researchers [22]–[23] widely recognize the existence of personality in dogs, but there has been little clarity regarding the nature and strength of personality consistency and the usefulness of ‘puppy tests’ in predicting adult behavior [29]–[30]. This lack of clarity is evidenced by a vote-count of prior results of personality consistency in dogs (Table 1), where 64.5% of studies report both positive and negative findings for personality consistency. Using meta-analysis, we quantitatively synthesized previous results to determine when personality may be consistent, the factors that influence personality consistency, and to provide recommendations regarding studies that will likely further the field.

Our results provide evidence for the broad proposition that dog personality is moderately consistent (overall average weighted effect size *r* = 0.43). This finding fits well with findings on the consistency of behavior in non-domesticated animals, where a recent meta-analyses reported an overall average weighted effect size of *r* = 0.37 [47] using fixed effects models (our fixed effect estimate was *r* = 0.29; see supporting information) for correlations of the same behavior through time. Another meta-analysis reported an overall average weighted effect size of 0.20 [87] using random effects models for correlations between different behaviors at the same time (i.e., a behavioral syndrome).

One factor that influenced personality consistency in dogs was age. Average weighted personality consistency estimates were different from zero in both puppies (*r* = 0.31) and adults (*r* = 0.51) and the adult dog personality consistency estimate was significantly greater than that observed in puppies. These results are in line with previous findings in dogs [27] and humans [49]. From a developmental perspective, increasing consistency or predictability may be observed with age if there are energetic or structural costs to changing one's personality [53]. Similarly, some theory suggests that strong personality consistency is to be expected when positive feedback loops exist between the individual and its environment [55], such as when individuals prefer to live in environments in which they perform at high levels [55], [88]. Most dogs do not select the environment in which they live in but dynamic social interactions with humans occur throughout ontogeny and could also in theory reinforce behaviors deemed appropriate by owners and other handlers. In theory, aging would allow for positive feedback loops to have a greater effect in adults than in puppies.

Another factor that influenced personality consistency in dogs was the interaction between personality dimension and age. In puppies, aggression and submissiveness were the most consistent dimensions, and neared estimates of personality consistency found in adults, while responsiveness to training and fearfulness were the least consistent dimensions (sociability consistency estimates in puppies was not different from zero). In adult dogs this was not the case; instead, all dimensions were equally consistent with the exception of submissiveness, where our analysis was restricted to a single study. Unfortunately, little is known concerning relative rates of development of different personality dimensions in puppies. However, one putative explanation for the observed differences in consistency among different personality dimensions in puppies may be proximate hormonal mechanisms. For example, androgens are known to influence both aggression [44] and submission [89] in other taxa. Corticosteroids are known to influence fearfulness [45]–[46]. Structurally, if different personality dimensions tend to be influenced by different proximate mechanisms, and these different underlying mechanisms have different rates of physical ontogeny (e.g., organ development, receptor density development, etc.), then different personality dimensions could end up having different rates of ‘fixation’, or observed consistency through time. From a practical perspective, it is worth noting that responsiveness to training was one of the least consistent personality dimensions observed in puppies, despite its importance to the general public [90]. These results imply that snapshots of responsiveness to training in puppies may not be an accurate assessment of a puppy's ability to respond or learn later training or obedience exercises [19]. Further studies are needed on the factors that influence consistency in all personality dimensions, but perhaps in particular responsiveness to training in puppies, because this dimension is especially relevant to human-dog relationships.

Time interval between tests was also found to play a small, but significant role on personality consistency estimates, with an overall negative relationship being observed. However, this effect of decreasing consistency with increasing interval was found mainly in adult dogs, where shorter (<10 weeks) time intervals tended to result in larger consistency estimates (*r* = 0.60) than longer (>24 weeks) time intervals (*r* = 0.32). All categorical time interval estimates were different from zero in adults but estimates of consistency fell from *r* = 0.60 over short intervals to *r* = 0.32 for long intervals. These smaller effect sizes for adult personality consistency over longer periods of adult life suggest that even as adults, personality dimensions are not fixed properties of individual dogs, but may instead be conducive to environmental and social manipulation and change.

Puppy consistency estimates for short, medium, and long test intervals were not different from one another, but were different from zero (*r* = 0.25 – 0.39). In addition, we found no difference in personality consistency estimates between dogs first tested as puppies and then either as puppies or adults during the second test period (*r* = 0.38 versus *r* = 0.40, respectively). One of the core questions facing many working and companion dog organizations is whether ‘puppy tests’ are predictive of later adult behavior [30]. Our results suggest that puppy personality is moderately consistent, and remains so, throughout the juvenile and into the adult period. This may especially true for particular personality dimensions, such as aggression or submissiveness, which appear to be as consistent as dimensions measured in adult dogs. Our results suggest that the blanket idea that ‘puppy tests do not work’ needs to be reconsidered – it may depend both on the personality dimension being considered as well as factors individual dogs experience throughout their life such as litter size [91]–[92], body mass, and early growth [93]. As in adults, puppy personality can be characterized as being both moderately consistent as well as sometimes highly plastic, depending on the personality dimension of interest.

We did not find a difference in personality consistency in working versus non-working dogs. We examined the possibility that (in)stability of the rearing environment might alter the consistency of personality, but we did not find evidence to support this idea one way or the other. Other studies have reported that some dog breeds have more consistent personalities than others [94], and it may be that consistency itself is also a ‘dimension’ that could be selected for, as opposed to selecting for absolute levels of behavior that are observed at any one time point. To our knowledge, no professional working dog programs or companion dog breeders have yet to investigate the possibility of selection for consistency per se, but our results suggest that personality consistency has not yet been altered by any artificial selection imposed by working dog programs.

Somewhat surprisingly, we did not find any differences in consistency estimates between behavioral codings versus behavioral ratings and between single versus aggregate measures. In principle, behavioral ratings and aggregate measures should yield more consistency because in both cases, error variance is reduced [61]. One explanation for this result may lie in the close social relationships dogs have shared with humans over the past 14,000 years [95]. In dogs, as opposed to other nonhuman animals, this relationship may have resulted in accurate perception of dog behavior by humans, regardless of the measurement method (i.e., error variance in coding methods and single measures are not different from ratings methods and aggregate measures). An alternative explanation somewhat supported by our results is that behavioral ratings yielded lower estimates of consistency than expected because observers using ratings methods had restricted relationships to the target subjects [60]. For 38% of the studies we were unable to determine the exact nature of the relationship between the rater and the subject but only 24% of studies that used ratings were from studies where longer-term knowledge of the dog could be implied (i.e., owners gave dogs' ratings). This issue reflects the current state of the literature (dog personality research is dominated by studies using working dogs [23], which use program staff with unknown personal relationships to the dogs to provide ratings).

It is encouraging that personality consistency estimates were moderate both when using single trait measures and aggregate trait measures. Again, this pattern was somewhat unintuitive, based on measurement theory. Our results could be explained if the close social ties between humans and dogs resulted in the researcher's ability to define single behavioral indicators (such as particular postures) that were precisely recognized by observers and that have strong ties to broader personality patterns. Alternatively, our results could have also occurred if aggregate measures consisted of behaviors that did not cohere and so should not have been combined together, thus decreasing aggregate measures' predictive validity [96]. Some analyses suggest that behaviors that are theoretically part of the same behavioral category may not be empirically related [56]. Unfortunately, dog personality studies often do not report measures of internal coherence of aggregate scales [96], so we were unable to test the idea that reliable aggregate measures had different consistency estimates than aggregate measures with unknown or low internal coherence reliability estimates. Nonetheless, the current results indicate that the choice of measurement method (i.e., behavioral codings versus behavioral ratings and single versus aggregate measures) may not be critical when determining the best way to measure consistent behavioral properties in dogs.

In principle, the greater the similarity between tests administered at different time points, the more consistent dog personality should appear because method variance is reduced. Our meta-analysis provided strong support for this pattern, showing that consistency was greater when testing instruments were identical across time points compared to when the two tests differed. Practical concerns drive many researchers to use different tests. For example, it is convenient for shelter staff to give behavioral assessments to dogs while in the shelter but exact follow up tests are not possible and instead are often conducted using a questionnaire that are given to the adoptive owner at a later date [e.g., 26, 59]. When these methods are used and the same behavior is measured in two different ways, personality may appear to be less consistent but the consistency estimate will be confounded with method variance. Of course, there are times when the same test cannot be given but our analyses suggest that efforts to create tests that are as conceptually similar as possible would be worthwhile. One potential issue with this moderator is that when the type of test differs between first and second assessments, so does the testing context. Thus, test type (e.g., exact same versus conceptually same behavioral assessments) is partially confounded in our analyses with test context (e.g., at a shelter vs. in the home). Future research is needed to separate the effects of these different factors.

### 

#### Limitations and Recommendations

Despite the obvious importance of understanding the consistency of personality in dogs, there is currently a paucity of studies examining the factors that influence personality consistency. Indeed, many pertinent research questions could not be addressed due to the small number of samples available for moderator analyses. For example, many dog personality studies focus on how well an earlier behavioral test can predict later ‘success’ or certification in a training program but ‘success’ is usually not well-defined (i.e., usually reported as a yes/no outcome) [97]. Attempts at defining explicit domains of success/failure (e.g., quantitative descriptions of behavior) could enable potential inclusion of these studies in future meta-analyses. Also, there could be differences based on training methods, but there were not enough studies to examine training differences based on different types of programs, shelters, or even by country. Finally, it would have been interesting to explore the role of moderators in a hierarchical fashion or simultaneously, but the lack of degrees of freedom limited our ability to do so.

Surprisingly, many studies did not even report the breeds used or individual breed results. There are differences in absolute levels of personality expression between breeds and breed clusters [98], so it is possible there are breed differences in personality consistency too. In addition, potentially important early environmental factors often go unreported. For example, across the 31 studies we examined, only 8 reported the weaning age of the dog and only 6 reported the age in which dogs were first housed singly. Early experiences, such as exposure to novel stimuli and socialization, are important factors in the biological development of dogs [99]–[100]. From a statistical standpoint, many studies did not report consistency in ways that could be translated to effect sizes (e.g., only mean level results were reported [101]) and many studies did not report confidence intervals. Effect size estimates and confidence intervals allow one to easily identify the relationship between tests and compare results across different samples, measures, and conditions [102]. We recommend effect sizes (*r* or *d*) and 95% confidence intervals are reported in all future dog personality studies. Changes in reporting practices could do much to alleviate this issue.

Currently, there are very few studies on personality development patterns. Just as some personality dimensions may be more or less consistent than others, some individuals' personalities may also be more or less consistent. Evidence from other taxa suggest that particular personality ‘types’ may be more or less capable of altering their behavior appropriately to environmental conditions [51], [103], and these personality by personality plasticity interactions may be widespread [104]. Indeed, the consistently moderate effect sizes for personality consistency witnessed here could be explained if some individuals were strongly consistent in their personality expression while others were not. Individual differences in consistency per se have important implications for a practical understanding of personality development and human-dog relationships; to our knowledge no dog studies have explicitly examined this aspect of personality development.

Little is known concerning the relationship between the development of proximate biological mechanisms (i.e., neuroendocrine systems) and the development of personality consistency in dogs. Some underlying biological mechanisms may have different rates of biological development [30]. There is a clear need to understand how differential rates of development of proximate mechanisms may impact the consistency of different personality dimensions. Likewise, studies on environmental effects that differentially affect personality dimension expression would be equally useful.

There are three other important limitations of any meta-analysis. First, moderator variables are likely confounded with one another in complex ways that make it difficult to tease apart the independent effects of each moderator. Second, it is possible that factors that are not examined also explain variability in effect size, perhaps better than those chosen in this meta-analysis. Third, synthesis-generated evidence should not be interpreted as supporting statements about causality [102]. When groups of effect sizes are compared within a research synthesis, the synthesis can only establish an association between a moderator variable and the outcomes of studies, not a causal connection. It is important that future research examine the factors that this meta-analysis has identified as influential in explaining differences in dog personality consistency using more controlled designs.

#### Summary

Taken together, our results indicate that personality is generally consistent in dogs. The question of personality consistency in dogs has implications for many areas of human society, and our meta-analysis is a first step towards quantitatively synthesizing the existing information on personality consistency in dogs. Along with the general theme of consistency observed here, some of the important factors that tended to influence consistency estimated in dogs include age, personality dimension, test interval, and the conceptual similarity between test situations. In puppies, the predictive validity of ‘puppy tests’ is most likely to be detected when measuring aggression and submissiveness and less so in other personality dimensions. These latter personality dimensions (responsiveness to training, fearfulness, activity, and sociability) may be more amenable to analyses of how, why, and when personality changes. In adult dogs, personality consistency was stronger than in puppies and equally predictable across all dimensions examined. Adult personality consistency estimates improved with a decreasing time interval between tests (which was not the case in puppies), but over longer time periods, personality in adults could be described as being moderately plastic. Our results suggest that useful future studies could examine the developmental rates of different proximate mechanisms underlying different personality dimensions, to address whether consistency per se can be viewed as a dimension in and of itself, and to identify the specific periods of life during which different personality dimensions stabilize. In addition, improved reporting methods are urgently needed to furnish researchers, working-dog organizations, breeders, shelters, and pet owners with the tools required to identify the factors likely to be responsible for personality predictability and change.

## Supporting Information

Figure S1 **PRISMA 2009 Flow Diagram.**
(DOC)

Supporting Information S1 **Results of Moderator Analysis using fixed effects models.**
(DOC)
