# Supplementary material for: Personality Consistency in Dogs: A Meta-Analysis
Source: PLoS One. 2013 Jan 23;8(1):e54907. doi: 10.1371/journal.pone.0054907 (PMC3553070; doi:10.1371/journal.pone.0054907)
Supplement: Figure S1 — PRISMA 2009 Flow Diagram. (DOC) [file pone.0054907.s001.doc]

Figure S1. PRISMA 2009 Flow Diagram


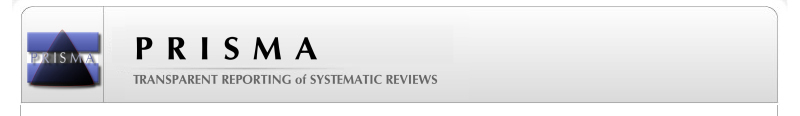


**Screening**

**Included**

**Eligibility**

**Identification**

Records identified through database searching
(n = 156)

Additional records identified through other sources
(n = 8)

Records after duplicates removed
(n = )

Records screened
(n = 164)

Records excluded
(n = 57)

Full-text articles assessed for eligibility
(n = 107)

Full-text articles excluded, with reasons
(n = 76)

Studies included in qualitative synthesis
(n = )

Studies included in quantitative synthesis (meta-analysis)
(n = 31)
