# Supplementary material for: Personality Consistency in Dogs: A Meta-Analysis
Source: PLoS One. 2013 Jan 23;8(1):e54907. doi: 10.1371/journal.pone.0054907 (PMC3553070; doi:10.1371/journal.pone.0054907)
Supplement: Supporting Information S1 — Results of Moderator Analysis using fixed effects models. (DOC) [file pone.0054907.s002.doc]

Supporting information

**Table S1. Results of Moderator Analysis using fixed effects models.**

| *Moderators* |  | *k* | *r* | *Lower* | *Higher* |
| --- | --- | --- | --- | --- | --- |
| **Jones and Gosling** | |  |  |  |  |
| Activity | | 12 | 0.32^**^ | 0.30 | 0.34 |
| Aggression | | 8 | 0.26^**^ | 0.24 | 0.28 |
| Fearfulness | | 22 | 0.26^**^ | 0.25 | 0.28 |
| Responsiveness to Training | | 11 | 0.22^**^ | 0.20 | 0.24 |
| Sociability | | 9 | 0.28^**^ | 0.26 | 0.32 |
| Submissiveness | | 4 | 0.42^**^ | 0.36 | 0.48 |
|  | |  |  |  |  |
| **Age at First Test** | |  |  |  |  |
|  | |  |  |  |  |
| Puppy | | 14 | 0.32^**^ | 0.31 | 0.33 |
| Adult | | 17 | 0.26^**^ | 0.25 | 0.27 |
|  | |  |  |  |  |
| **Role of Dog** | |  |  |  |  |
|  | |  |  |  |  |
| Companion | | 15 | 0.27^**^ | 0.26 | 0.28 |
| Working | | 10 | 0.34^**^ | 0.33 | 0.35 |
|  | |  |  |  |  |
| **Measurement Method** | |  |  |  |  |
|  | |  |  |  |  |
| Codings | | 8 | 0.29^**^ | 0.28 | 0.31 |
| Ratings | | 21 | 0.29^**^ | 0.28 | 0.30 |
|  | |  |  |  |  |
| **Measurement** | |  |  |  |  |
|  | |  |  |  |  |
| Single | | 16 | 0.34^**^ | 0.33 | 0.35 |
| Aggregate | | 20 | 0.34^**^ | 0.33 | 0.35 |
|  | |  |  |  |  |
| **Test Two** | |  |  |  |  |
|  | |  |  |  |  |
| Same | | 22 | 0.36^**^ | 0.35 | 0.37 |
| Different | | 16 | 0.14^**^ | 0.13 | 0.15 |
| **Overall Estimate** | | 31 | 0.29^**^ | 0.29 | 0.30 |

^+^p < 0.10, ^*^P < 0.05, ^**^p < 0.01

**Table S2. Contrasts (Q-values) for Jones and Gosling (2005) personality dimensions in puppies using fixed effects models.**

|  | Aggression | | Fearfulness | Responsiveness | Sociability | Submissiveness |  |
| --- | --- | --- | --- | --- | --- | --- | --- |
| Activity | | 15.89^**^ | 14.67^**^ | 42.47^**^ | 3.51^+^ | 8.57^**^ |  |
| Aggression | |  | 0.39 | 7.94^**^ | 2.39 | 21.60^**^ |  |
| Fearfulness | |  |  | 14.52^**^ | 1.41 | 20.33^**^ |  |
| Responsiveness to Training | |  |  |  | 14.38^**^ | 32.41^**^ |  |
| Sociability | |  |  |  |  | 14.27^**^ |  |

^+^*p* < .10, **p* < .05, ** *p* < .01, all values are Q values

**Table S3. Interaction of personality factors and age of dog using fixed effects models.**

|  |  |  | 95% Confidence Interval | |
| --- | --- | --- | --- | --- |
|  | *k* | *r* | *Low Estimate* | *High Estimate* |
| **Puppy** |  |  |  |  |
|  |  |  |  |  |
| Activity | 7 | 0.34^**^ | 0.31 | 0.37 |
| Aggression | 2 | 0.55^**^ | 0.51 | 0.59 |
| Fearfulness | 10 | 0.29^**^ | 0.28 | 0.31 |
| Responsiveness to Training | 6 | 0.19^**^ | 0.17 | 0.21 |
| Sociability | 4 | 0.38^**^ | 0.35 | 0.42 |
| Submissiveness | 3 | 0.43^**^ | 0.36 | 0.49 |
|  |  |  |  |  |
| **Adult** |  |  |  |  |
|  |  |  |  |  |
| Activity | 7 | 0.29^**^ | 0.25 | 0.33 |
| Aggression | 6 | 0.20^**^ | 0.18 | 0.22 |
| Fearfulness | 13 | 0.18^**^ | 0.15 | 0.21 |
| Responsiveness to Training | 4 | 0.29^**^ | 0.25 | 0.32 |
| Sociability | 6 | 0.15^**^ | 0.10 | 0.19 |
| Submissiveness | 1 | -0.13 | -0.73 | 0.59 |

^+^p < 0.10, ^*^p < 0.05, ^**^p < 0.01

**Table S4. Contrasts (Q-values) for Jones and Gosling (2005) personality dimensions and age interaction using fixed effects models.**

|  | |  | Aggression | Fearfulness | Responsiveness | Sociability | Submissiveness |  |
| --- | --- | --- | --- | --- | --- | --- | --- | --- |
| Puppy |  | |  |  |  |  |  |  |
|  | Activity | | 67.87^**^ | 6.28^**^ | 56.49^**^ | 3.61^+^ | 5.92^*^ |  |
|  | Aggression | |  | 116.22^**^ | 195.84^**^ | 39.64^**^ | 12.10^**^ |  |
|  | Fearfulness | |  |  | 49.15^**^ | 19.23^**^ | 14.45^**^ |  |
|  | Responsiveness to Training | |  |  |  | 75.36^**^ | 40.89^**^ |  |
|  | Sociability | |  |  |  |  | 1.42 |  |
| Adult |  | |  |  |  |  |  |  |
|  | Activity | | 16.84^**^ | 21.78^**^ | 0.03 | 23.04^**^ | 1.08 |  |
|  | Aggression | |  | 1.41 | 15.64^**^ | 4.17^*^ | 0.65 |  |
|  | Fearfulness | |  |  | 20.56^**^ | 1.22 | 0.56 |  |
|  | Responsiveness to Training | |  |  |  | 21.92^**^ | 1.05 |  |
|  | Sociability | |  |  |  |  | 0.45 |  |

^+^*p* < .10, **p* < .05, ** *p* < .01, all values are Q values

**Table S5. Fixed effects meta-regression for ‘time interval between tests’ moderator.**

| *Variable* | *B* | *P* |
| --- | --- | --- |
| Interval | -0.003 | <0.001 |
| Regression constant | 0.41 |  |
| Overall model | Q(1) = 629.20 |  |
| Residual | Q(818) = 5833.59 |  |
| Total | Q(819) = 6522.80 |  |
| R^2^ = 0.11 |  |  |
